# Bis(2,4,6-trimethyl­anilinium) sulfate monohydrate

**DOI:** 10.1107/S1600536811038086

**Published:** 2011-09-30

**Authors:** Tao Rong

**Affiliations:** a Ordered Matter Science Research Center, Southeast University, Nanjing 210096, People’s Republic of China

## Abstract

In the crystal structure of the title compound, 2C_9_H_14_N^+^·SO_4_^2−^·H_2_O, the components are linked by inter­molecular N—H⋯O and O—H⋯O hydrogen bonds. N—H⋯S and O—H⋯S inter­actions also occur.

## Related literature

The title compound was obtained during attempts to obtain potential ferroelectric phase-transition materials. For general background to ferroelectric organic frameworks, see: Ye *et al.* (2006 ▶, 2009 ▶); Fu *et al.* (2007 ▶) and for phase transition of ferroelectric materials, see: Zhang *et al.* (2008 ▶); Zhao *et al.* (2008 ▶).
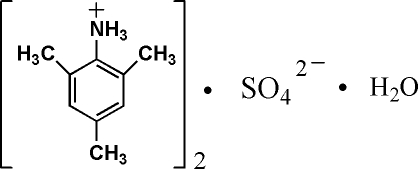


## Experimental

### 

#### Crystal data

2C_9_H_14_N^+^·SO_4_^2−^·H_2_O
*M* *_r_* = 386.50
Orthorhombic, 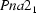

*a* = 7.7414 (12) Å
*b* = 30.418 (5) Å
*c* = 16.949 (3) Å
*V* = 3991.3 (11) Å^3^
*Z* = 8
Mo *K*α radiation
μ = 0.19 mm^−1^
*T* = 293 K
0.20 × 0.20 × 0.20 mm

#### Data collection

Rigaku SCXmini diffractometer
Absorption correction: multi-scan (*CrystalClear*; Rigaku, 2005 ▶) *T*_min_ = 0.962, *T*_max_ = 0.962
41971 measured reflections
9160 independent reflections
7532 reflections with *I* > 2σ(*I*)
*R*_int_ = 0.072

#### Refinement

*R*[*F* ^2^ > 2σ(*F* ^2^)] = 0.061
*wR*(*F* ^2^) = 0.143
*S* = 1.06
9160 reflections
485 parameters
411 restraints
H-atom parameters constrained
Δρ_max_ = 0.21 e Å^−3^
Δρ_min_ = −0.41 e Å^−3^
Absolute structure: Flack (1983 ▶), 4422 Friedel pairs
Flack parameter: 0.05 (9)

### 

Data collection: *CrystalClear* (Rigaku, 2005 ▶); cell refinement: *CrystalClear*; data reduction: *CrystalClear*; program(s) used to solve structure: *SHELXS97* (Sheldrick, 2008 ▶); program(s) used to refine structure: *SHELXL97* (Sheldrick, 2008 ▶); molecular graphics: *DIAMOND* (Brandenburg & Putz, 2005 ▶); software used to prepare material for publication: *SHELXL97*.

## Supplementary Material

Crystal structure: contains datablock(s) I, global. DOI: 10.1107/S1600536811038086/zk2028sup1.cif

Structure factors: contains datablock(s) I. DOI: 10.1107/S1600536811038086/zk2028Isup2.hkl

Supplementary material file. DOI: 10.1107/S1600536811038086/zk2028Isup3.cml

Additional supplementary materials:  crystallographic information; 3D view; checkCIF report

## Figures and Tables

**Table 1:** Hydrogen-bond geometry (Å, °)

*D*—H⋯*A*	*D*—H	H⋯*A*	*D*⋯*A*	*D*—H⋯*A*
N1—H1*D*⋯O6	0.89	1.81	2.668 (4)	162
N1—H1*D*⋯S1	0.89	2.74	3.603 (3)	164
N2—H2*B*⋯O1*W*	0.89	2.12	2.834 (4)	137
N2—H2*C*⋯O4	0.89	1.88	2.763 (4)	173
N2—H2*C*⋯S2	0.89	3.00	3.861 (3)	162
N3—H3*A*⋯O7	0.89	1.89	2.761 (4)	166
N3—H3*B*⋯O2*W*	0.89	2.12	2.877 (4)	142
N4—H4*A*⋯O7	0.89	1.97	2.800 (4)	155
N3—H3*A*⋯S1	0.89	3.05	3.858 (3)	153
O1*W*—H1*WA*⋯O1	0.78	2.02	2.782 (4)	165
O1*W*—H1*WB*⋯O5	0.97	1.82	2.788 (4)	173
O1*W*—H1*WB*⋯S1	0.97	2.88	3.805 (3)	159
O2*W*—H2*WB*⋯O5	0.80	1.97	2.756 (4)	166
O2*W*—H2*WB*⋯S1	0.80	2.99	3.761 (3)	162
N1—H1*E*⋯O2*W*^i^	0.89	1.99	2.837 (4)	158
N1—H1*F*⋯O4^ii^	0.89	1.99	2.807 (4)	153
N1—H1*F*⋯S2^ii^	0.89	2.92	3.622 (3)	137
N2—H2*A*⋯O6^iii^	0.89	1.85	2.735 (4)	171
N2—H2*A*⋯S1^iii^	0.89	3.01	3.800 (3)	149
N3—H3*C*⋯O2^iv^	0.89	1.81	2.695 (4)	178
N3—H3*C*⋯S2^iv^	0.89	2.96	3.788 (3)	156
N4—H4*B*⋯O1*W*^iv^	0.89	1.97	2.842 (4)	165
N4—H4*C*⋯O2^ii^	0.89	1.79	2.683 (4)	178
N4—H4*C*⋯S2^ii^	0.89	2.81	3.616 (3)	151
O2*W*—H2*WA*⋯O1^v^	0.94	1.86	2.781 (4)	168
O2*W*—H2*WA*⋯S2^v^	0.94	2.88	3.791 (3)	165

Symmetry codes: (i) 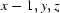; (ii) 
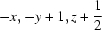
; (iii) 
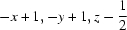
; (iv) 
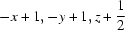
; (v) 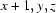.

**Fig. 1.**
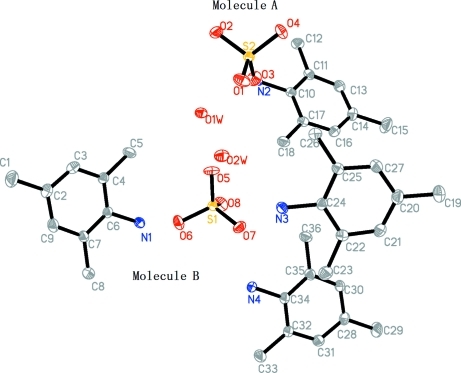
The molecular structure of the title compound, with the atomic numbering scheme.Displacement ellipsoids are drawn at the 30%

**Fig. 2.**
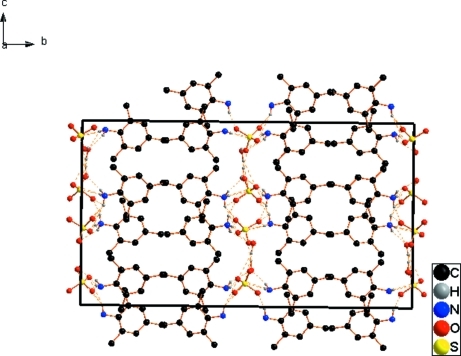
A view of the packing of the title compound, stacking along the a axis. Dashed lines indicate hydrogen bonds.

## supplementary crystallographic information

### Comment

The study of ferroelectric materials has received much attention and some materials have predominantly dielectric–ferroelectric performance (Ye *et al.*, 2006; Fu *et al.*, 2007; Zhao *et al.* 2008; Zhang *et al.*, 2008; Ye *et al.*, 2009), As a part of our work to obtain potential ferroelectric phase-transitionmaterials, we report herein on the crystal structure of title compound. Unluckily, the title compound has no dielectric anomalies in the temperature range 93–453 K, suggesting that it might be only a paraelectric.

The asymmetric unit of the title compound is is shown in Fig. 1. There are two independent molecules [labelled A & B]. The crystal packing (Fig. 2) is stabilized by weak intermolecular N—H···O and O—H···O hydrogen bonds between the [C_9_H_14_N]^+^ cations and, SO_4_^2-^ anions and H_2_O (see; Table 1).

### Experimental

For the preparation of the title compound, the water solution ofthe sulfuric acid(1 g) was added to the ethanol solution of the 2,4,6-trimethylaniline, The resulting precipitate was filtered. Colorless crystals suitable for X-ray analysis were formed after several weeks by slow evaporation of the solvent at room temperature.

### Refinement

Positional parameters of all the H atoms bonded to C atoms were calculated geometrically and were allowed to ride on the C atoms to which they are bonded, with *U*_iso_(H) = 1.2Ueq(C) and *U*_iso_(H) = 1.5Ueq(C) for the methyl group. The other H bonded to O/N atoms were calculated geometrically and were allowed to ride on the O/N atoms with *U*_iso_(H) = 1.2Ueq(N) and *U*_iso_(H) = 1.5Ueq(O).

### Crystal data

**Table:**

2C_9_H_14_N^+^·SO_4_^2^^−^·H_2_O	*F*(000) = 1664
*M**_r_* = 386.50	*D*_x_ = 1.286 Mg m^−^^3^
Orthorhombic, *P**n**a*2_1_	Mo *K*α radiation, λ = 0.71073 Å
Hall symbol: P 2c -2n	Cell parameters from 9163 reflections
*a* = 7.7414 (12) Å	θ = 2.3–27.5°
*b* = 30.418 (5) Å	µ = 0.19 mm^−^^1^
*c* = 16.949 (3) Å	*T* = 293 K
*V* = 3991.3 (11) Å^3^	Prism, colourless
*Z* = 8	0.20 × 0.20 × 0.20 mm

### Data collection

**Table:**

Rigaku SCXmini diffractometer	9160 independent reflections
Radiation source: fine-focus sealed tube	7532 reflections with *I* > 2σ(*I*)
graphite	*R*_int_ = 0.072
Detector resolution: 13.6612 pixels mm^-1^	θ_max_ = 27.5°, θ_min_ = 2.7°
CCD_Profile_fitting scans	*h* = −10→10
Absorption correction: multi-scan (*CrystalClear*; Rigaku, 2005)	*k* = −39→39
*T*_min_ = 0.962, *T*_max_ = 0.962	*l* = −22→22
41971 measured reflections	

### Refinement

**Table:**

Refinement on *F*^2^	Secondary atom site location: difference Fourier map
Least-squares matrix: full	Hydrogen site location: inferred from neighbouring sites
*R*[*F*^2^ > 2σ(*F*^2^)] = 0.061	H-atom parameters constrained
*wR*(*F*^2^) = 0.143	*w* = 1/[σ^2^(*F*_o_^2^) + (0.0603*P*)^2^ + 0.9745*P*] where *P* = (*F*_o_^2^ + 2*F*_c_^2^)/3
*S* = 1.06	(Δ/σ)_max_ = 0.061
9160 reflections	Δρ_max_ = 0.21 e Å^−^^3^
485 parameters	Δρ_min_ = −0.41 e Å^−^^3^
411 restraints	Absolute structure: Flack (1983), 4422 Friedel pairs
Primary atom site location: structure-invariant direct methods	Flack parameter: 0.05 (9)

### Special details

**Table:**

Refinement. Refinement of *F*^2^ against ALL reflections. The weighted *R*-factor *wR* and goodness of fit *S* are based on *F*^2^, conventional *R*-factors *R* are based on *F*, with *F* set to zero for negative *F*^2^. The threshold expression of *F*^2^ > σ(*F*^2^) is used only for calculating *R*-factors(gt) *etc*. and is not relevant to the choice of reflections for refinement. *R*-factors based on *F*^2^ are statistically about twice as large as those based on *F*, and *R*- factors based on ALL data will be even larger.

### Fractional atomic coordinates and isotropic or equivalent isotropic displacement parameters (Å^2^)

**Table:**

	*x*	*y*	*z*	*U*_iso_*/*U*_eq_	
C1	0.3645 (7)	0.74678 (12)	0.3953 (3)	0.0612 (12)	
H1A	0.2698	0.7659	0.3825	0.092*	
H1B	0.4454	0.7465	0.3524	0.092*	
H1C	0.4208	0.7572	0.4421	0.092*	
C2	0.2968 (5)	0.70050 (11)	0.4092 (2)	0.0422 (8)	
C3	0.2874 (5)	0.67005 (12)	0.3479 (2)	0.0398 (8)	
H3D	0.3223	0.6784	0.2976	0.048*	
C4	0.2272 (4)	0.62748 (12)	0.3598 (2)	0.0326 (8)	
C5	0.2256 (6)	0.59543 (13)	0.2924 (3)	0.0464 (11)	
H5A	0.1090	0.5912	0.2746	0.070*	
H5B	0.2723	0.5678	0.3097	0.070*	
H5C	0.2944	0.6067	0.2499	0.070*	
C6	0.1750 (4)	0.61570 (11)	0.4357 (2)	0.0290 (7)	
C7	0.1817 (5)	0.64510 (11)	0.4987 (2)	0.0333 (7)	
C8	0.1295 (5)	0.63270 (12)	0.5813 (2)	0.0421 (9)	
H8A	0.1720	0.6544	0.6177	0.063*	
H8B	0.1774	0.6045	0.5943	0.063*	
H8C	0.0058	0.6313	0.5846	0.063*	
C9	0.2440 (5)	0.68731 (12)	0.4831 (2)	0.0411 (8)	
H9A	0.2501	0.7074	0.5244	0.049*	
C10	0.4137 (4)	0.39188 (11)	0.1167 (2)	0.0300 (7)	
C11	0.3603 (4)	0.36232 (11)	0.0583 (2)	0.0345 (8)	
C12	0.3073 (5)	0.37656 (13)	−0.0227 (2)	0.0425 (9)	
H12A	0.4049	0.3749	−0.0576	0.064*	
H12B	0.2659	0.4063	−0.0208	0.064*	
H12C	0.2173	0.3576	−0.0417	0.064*	
C13	0.3624 (5)	0.31764 (12)	0.0775 (2)	0.0432 (8)	
H13A	0.3264	0.2974	0.0398	0.052*	
C14	0.4161 (5)	0.30232 (12)	0.1505 (2)	0.0425 (9)	
C15	0.4195 (7)	0.25288 (12)	0.1675 (3)	0.0642 (13)	
H15A	0.3071	0.2406	0.1580	0.096*	
H15B	0.4511	0.2481	0.2216	0.096*	
H15C	0.5024	0.2389	0.1337	0.096*	
C16	0.4709 (5)	0.33302 (12)	0.2059 (2)	0.0395 (8)	
H16A	0.5101	0.3230	0.2545	0.047*	
C17	0.4699 (4)	0.37818 (12)	0.1919 (2)	0.0324 (8)	
C18	0.5313 (5)	0.40923 (14)	0.2532 (2)	0.0410 (9)	
H18A	0.4362	0.4268	0.2713	0.062*	
H18B	0.6187	0.4280	0.2313	0.062*	
H18C	0.5786	0.3930	0.2967	0.062*	
C19	1.0994 (7)	0.25935 (12)	0.3873 (3)	0.0590 (12)	
H19A	1.1506	0.2482	0.4348	0.088*	
H19B	1.1821	0.2579	0.3451	0.088*	
H19C	1.0000	0.2420	0.3740	0.088*	
C20	1.0455 (5)	0.30644 (11)	0.3998 (2)	0.0420 (8)	
C21	0.9827 (5)	0.32054 (12)	0.4715 (2)	0.0415 (8)	
H21A	0.9691	0.3000	0.5116	0.050*	
C22	0.9385 (4)	0.36406 (11)	0.4870 (2)	0.0334 (7)	
C23	0.8725 (6)	0.37680 (13)	0.5670 (2)	0.0445 (9)	
H23A	0.8207	0.3517	0.5918	0.067*	
H23B	0.7879	0.3997	0.5616	0.067*	
H23C	0.9668	0.3872	0.5987	0.067*	
C24	0.9606 (4)	0.39414 (11)	0.4253 (2)	0.0296 (7)	
C25	1.0192 (4)	0.38128 (13)	0.3507 (2)	0.0323 (8)	
C26	1.0393 (5)	0.41393 (14)	0.2835 (2)	0.0409 (9)	
H26A	1.0863	0.3992	0.2382	0.061*	
H26B	1.1159	0.4371	0.2994	0.061*	
H26C	0.9286	0.4261	0.2704	0.061*	
C27	1.0603 (5)	0.33733 (12)	0.3397 (2)	0.0390 (8)	
H27A	1.0990	0.3282	0.2904	0.047*	
C28	0.3445 (5)	0.29434 (11)	0.6463 (2)	0.0416 (9)	
C29	0.3301 (7)	0.24593 (12)	0.6662 (3)	0.0656 (13)	
H29A	0.2923	0.2427	0.7198	0.098*	
H29B	0.4408	0.2322	0.6598	0.098*	
H29C	0.2480	0.2323	0.6316	0.098*	
C30	0.3880 (5)	0.30767 (11)	0.5709 (2)	0.0405 (8)	
H30A	0.4075	0.2864	0.5325	0.049*	
C31	0.3125 (5)	0.32664 (11)	0.7020 (2)	0.0399 (8)	
H31A	0.2792	0.3181	0.7525	0.048*	
C32	0.3279 (5)	0.37103 (12)	0.6857 (2)	0.0341 (8)	
C33	0.2834 (6)	0.40487 (14)	0.7471 (3)	0.0483 (11)	
H33A	0.2361	0.3905	0.7927	0.072*	
H33B	0.1998	0.4250	0.7259	0.072*	
H33C	0.3857	0.4207	0.7618	0.072*	
C34	0.3768 (4)	0.38316 (11)	0.6097 (2)	0.0308 (7)	
C35	0.4038 (4)	0.35176 (11)	0.5502 (2)	0.0346 (8)	
C36	0.4449 (5)	0.36414 (13)	0.4659 (2)	0.0442 (9)	
H36A	0.5589	0.3765	0.4633	0.066*	
H36B	0.3624	0.3854	0.4475	0.066*	
H36C	0.4397	0.3384	0.4332	0.066*	
N1	0.1239 (4)	0.56985 (9)	0.44948 (18)	0.0305 (7)	
H1D	0.2179	0.5530	0.4519	0.046*	
H1E	0.0568	0.5608	0.4101	0.046*	
H1F	0.0662	0.5680	0.4948	0.046*	
N2	0.4050 (4)	0.43893 (9)	0.09915 (18)	0.0321 (7)	
H2A	0.4576	0.4442	0.0534	0.048*	
H2B	0.4572	0.4540	0.1374	0.048*	
H2C	0.2950	0.4472	0.0959	0.048*	
N3	0.9247 (4)	0.44069 (9)	0.44031 (18)	0.0298 (6)	
H3A	0.8114	0.4446	0.4460	0.045*	
H3B	0.9622	0.4567	0.3998	0.045*	
H3C	0.9786	0.4491	0.4842	0.045*	
N4	0.3946 (4)	0.43013 (8)	0.59140 (18)	0.0299 (6)	
H4A	0.4559	0.4333	0.5474	0.045*	
H4B	0.4483	0.4436	0.6310	0.045*	
H4C	0.2904	0.4419	0.5847	0.045*	
O1	0.0627 (4)	0.50945 (10)	0.19281 (18)	0.0436 (7)	
O2	−0.0796 (3)	0.53415 (9)	0.07533 (17)	0.0415 (6)	
O3	−0.1988 (3)	0.47421 (9)	0.14906 (19)	0.0449 (7)	
O4	0.0670 (3)	0.46503 (9)	0.07558 (17)	0.0392 (6)	
O5	0.5726 (4)	0.50569 (10)	0.34540 (17)	0.0414 (7)	
O6	0.4401 (3)	0.53554 (9)	0.46156 (18)	0.0432 (7)	
O7	0.5875 (3)	0.46616 (9)	0.46752 (18)	0.0402 (6)	
O8	0.3141 (3)	0.47293 (9)	0.39917 (19)	0.0453 (7)	
O1W	0.4214 (3)	0.51438 (8)	0.19692 (17)	0.0345 (6)	
H1WA	0.3220	0.5173	0.1994	0.052*	
H1WB	0.4665	0.5127	0.2502	0.052*	
O2W	0.9251 (3)	0.51838 (9)	0.34350 (16)	0.0374 (6)	
H2WB	0.8230	0.5176	0.3506	0.056*	
H2WA	0.9567	0.5133	0.2910	0.056*	
S1	0.47640 (10)	0.49460 (3)	0.41718 (5)	0.02659 (19)	
S2	−0.03898 (10)	0.49501 (3)	0.12443 (5)	0.02667 (19)	

### Atomic displacement parameters (Å^2^)

**Table:**

	*U*^11^	*U*^22^	*U*^33^	*U*^12^	*U*^13^	*U*^23^
C1	0.068 (3)	0.041 (2)	0.074 (3)	−0.015 (2)	0.005 (3)	0.001 (2)
C2	0.044 (2)	0.0324 (18)	0.050 (2)	−0.0027 (14)	−0.0001 (18)	0.0047 (16)
C3	0.0382 (19)	0.042 (2)	0.040 (2)	−0.0005 (16)	0.0010 (16)	0.0055 (16)
C4	0.0319 (17)	0.0317 (18)	0.0341 (18)	0.0042 (14)	−0.0004 (15)	0.0032 (15)
C5	0.065 (3)	0.041 (2)	0.033 (2)	−0.009 (2)	0.009 (2)	−0.0054 (18)
C6	0.0268 (16)	0.0287 (16)	0.0315 (17)	0.0058 (12)	−0.0047 (13)	0.0010 (13)
C7	0.0335 (18)	0.0309 (17)	0.0354 (18)	0.0019 (14)	−0.0049 (15)	−0.0026 (14)
C8	0.054 (2)	0.038 (2)	0.0345 (19)	0.0017 (17)	−0.0015 (18)	−0.0039 (16)
C9	0.046 (2)	0.0325 (18)	0.045 (2)	−0.0008 (15)	−0.0051 (17)	−0.0067 (15)
C10	0.0279 (16)	0.0314 (17)	0.0307 (18)	0.0017 (12)	0.0055 (14)	−0.0004 (14)
C11	0.0295 (17)	0.0394 (19)	0.0347 (18)	0.0018 (14)	0.0031 (14)	−0.0058 (15)
C12	0.050 (2)	0.046 (2)	0.0315 (19)	−0.0019 (17)	−0.0078 (17)	−0.0013 (17)
C13	0.050 (2)	0.0359 (18)	0.043 (2)	−0.0035 (16)	−0.0014 (18)	−0.0048 (16)
C14	0.047 (2)	0.0313 (18)	0.050 (2)	0.0014 (15)	0.0009 (18)	0.0020 (16)
C15	0.088 (3)	0.030 (2)	0.074 (3)	0.001 (2)	−0.013 (3)	0.005 (2)
C16	0.049 (2)	0.0341 (18)	0.0351 (19)	−0.0001 (16)	−0.0019 (17)	0.0060 (15)
C17	0.0322 (17)	0.0359 (18)	0.0291 (18)	0.0003 (14)	0.0048 (14)	−0.0028 (14)
C18	0.051 (2)	0.042 (2)	0.030 (2)	−0.0041 (17)	−0.0009 (17)	−0.0020 (17)
C19	0.071 (3)	0.033 (2)	0.073 (3)	0.009 (2)	0.001 (2)	−0.001 (2)
C20	0.050 (2)	0.0302 (17)	0.046 (2)	0.0014 (15)	−0.0038 (18)	−0.0017 (15)
C21	0.051 (2)	0.0302 (17)	0.043 (2)	−0.0030 (16)	0.0020 (18)	0.0052 (15)
C22	0.0336 (18)	0.0357 (17)	0.0308 (17)	0.0009 (14)	0.0014 (14)	0.0000 (14)
C23	0.055 (2)	0.043 (2)	0.036 (2)	−0.0069 (18)	0.0055 (18)	0.0069 (17)
C24	0.0301 (16)	0.0288 (16)	0.0298 (17)	0.0010 (12)	−0.0031 (14)	−0.0005 (14)
C25	0.0286 (16)	0.0361 (19)	0.0323 (19)	0.0013 (14)	−0.0013 (14)	−0.0049 (14)
C26	0.050 (2)	0.043 (2)	0.031 (2)	0.0033 (17)	0.0027 (17)	0.0009 (17)
C27	0.043 (2)	0.0372 (19)	0.0370 (19)	0.0027 (16)	0.0017 (16)	−0.0062 (15)
C28	0.055 (2)	0.0257 (17)	0.045 (2)	−0.0022 (15)	0.0002 (18)	0.0029 (14)
C29	0.099 (4)	0.0261 (19)	0.072 (3)	−0.003 (2)	0.017 (3)	0.0068 (19)
C30	0.058 (2)	0.0249 (16)	0.0390 (19)	0.0001 (16)	0.0037 (17)	−0.0044 (14)
C31	0.051 (2)	0.0337 (18)	0.0346 (18)	−0.0039 (16)	0.0059 (17)	0.0035 (15)
C32	0.0366 (18)	0.0298 (17)	0.0358 (19)	−0.0003 (14)	−0.0005 (15)	−0.0010 (15)
C33	0.064 (3)	0.042 (2)	0.038 (3)	0.001 (2)	0.018 (2)	−0.0027 (19)
C34	0.0280 (16)	0.0281 (17)	0.0363 (19)	0.0013 (13)	−0.0013 (14)	0.0008 (14)
C35	0.0370 (18)	0.0305 (17)	0.0363 (18)	−0.0012 (15)	0.0022 (15)	−0.0033 (14)
C36	0.062 (3)	0.041 (2)	0.0300 (18)	−0.0044 (18)	0.0017 (18)	−0.0074 (16)
N1	0.0279 (14)	0.0328 (16)	0.0307 (17)	0.0043 (12)	−0.0001 (12)	−0.0036 (13)
N2	0.0334 (15)	0.0336 (16)	0.0291 (17)	0.0037 (12)	0.0009 (12)	−0.0020 (13)
N3	0.0323 (15)	0.0289 (15)	0.0284 (16)	0.0012 (11)	0.0003 (12)	−0.0014 (12)
N4	0.0351 (15)	0.0252 (15)	0.0295 (16)	0.0026 (11)	−0.0003 (12)	−0.0001 (12)
O1	0.0407 (15)	0.0633 (19)	0.0269 (15)	−0.0010 (12)	−0.0036 (12)	−0.0122 (14)
O2	0.0436 (14)	0.0446 (15)	0.0363 (15)	0.0127 (12)	0.0077 (12)	0.0149 (13)
O3	0.0324 (13)	0.0462 (16)	0.056 (2)	−0.0073 (12)	0.0056 (12)	0.0068 (14)
O4	0.0365 (13)	0.0436 (15)	0.0376 (15)	0.0099 (11)	−0.0022 (12)	−0.0128 (13)
O5	0.0392 (13)	0.0624 (19)	0.0226 (14)	−0.0029 (12)	0.0020 (12)	0.0061 (13)
O6	0.0458 (15)	0.0353 (14)	0.0484 (18)	0.0093 (12)	−0.0113 (13)	−0.0150 (13)
O7	0.0383 (13)	0.0424 (15)	0.0397 (16)	0.0113 (11)	0.0005 (12)	0.0148 (13)
O8	0.0312 (13)	0.0518 (17)	0.0529 (18)	−0.0075 (12)	−0.0033 (12)	−0.0133 (15)
O1W	0.0344 (13)	0.0412 (15)	0.0281 (14)	−0.0010 (10)	−0.0020 (11)	−0.0006 (12)
O2W	0.0349 (12)	0.0493 (17)	0.0279 (15)	0.0004 (12)	−0.0016 (11)	0.0034 (13)
S1	0.0277 (4)	0.0280 (4)	0.0240 (5)	0.0017 (3)	−0.0003 (4)	−0.0003 (4)
S2	0.0277 (4)	0.0291 (4)	0.0232 (4)	0.0018 (3)	0.0005 (3)	0.0010 (4)

### Geometric parameters (Å, °)

**Table:**

C1—C2	1.521 (5)	C23—H23C	0.9600
C1—H1A	0.9600	C24—C25	1.400 (5)
C1—H1B	0.9600	C24—N3	1.465 (4)
C1—H1C	0.9600	C25—C27	1.387 (5)
C2—C9	1.377 (5)	C25—C26	1.519 (5)
C2—C3	1.394 (5)	C26—H26A	0.9600
C3—C4	1.391 (5)	C26—H26B	0.9600
C3—H3D	0.9300	C26—H26C	0.9600
C4—C6	1.396 (5)	C27—H27A	0.9300
C4—C5	1.501 (5)	C28—C30	1.382 (5)
C5—H5A	0.9600	C28—C31	1.385 (5)
C5—H5B	0.9600	C28—C29	1.515 (5)
C5—H5C	0.9600	C29—H29A	0.9600
C6—C7	1.393 (5)	C29—H29B	0.9600
C6—N1	1.468 (4)	C29—H29C	0.9600
C7—C9	1.397 (5)	C30—C35	1.392 (4)
C7—C8	1.506 (5)	C30—H30A	0.9300
C8—H8A	0.9600	C31—C32	1.384 (5)
C8—H8B	0.9600	C31—H31A	0.9300
C8—H8C	0.9600	C32—C34	1.392 (5)
C9—H9A	0.9300	C32—C33	1.503 (5)
C10—C11	1.400 (5)	C33—H33A	0.9600
C10—C17	1.410 (5)	C33—H33B	0.9600
C10—N2	1.463 (4)	C33—H33C	0.9600
C11—C13	1.398 (5)	C34—C35	1.405 (5)
C11—C12	1.497 (5)	C34—N4	1.469 (4)
C12—H12A	0.9600	C35—C36	1.512 (5)
C12—H12B	0.9600	C36—H36A	0.9600
C12—H12C	0.9600	C36—H36B	0.9600
C13—C14	1.386 (5)	C36—H36C	0.9600
C13—H13A	0.9300	N1—H1D	0.8900
C14—C16	1.390 (5)	N1—H1E	0.8900
C14—C15	1.531 (5)	N1—H1F	0.8900
C15—H15A	0.9600	N2—H2A	0.8900
C15—H15B	0.9600	N2—H2B	0.8900
C15—H15C	0.9600	N2—H2C	0.8900
C16—C17	1.394 (5)	N3—H3A	0.8900
C16—H16A	0.9300	N3—H3B	0.8900
C17—C18	1.482 (5)	N3—H3C	0.8900
C18—H18A	0.9600	N4—H4A	0.8900
C18—H18B	0.9600	N4—H4B	0.8900
C18—H18C	0.9600	N4—H4C	0.8900
C19—C20	1.507 (5)	O1—S2	1.468 (3)
C19—H19A	0.9600	O2—S2	1.486 (3)
C19—H19B	0.9600	O3—S2	1.451 (3)
C19—H19C	0.9600	O4—S2	1.480 (3)
C20—C21	1.378 (5)	O5—S1	1.466 (3)
C20—C27	1.391 (5)	O6—S1	1.482 (3)
C21—C22	1.392 (5)	O7—S1	1.489 (3)
C21—H21A	0.9300	O8—S1	1.451 (2)
C22—C24	1.399 (5)	O1W—H1WA	0.7755
C22—C23	1.500 (5)	O1W—H1WB	0.9693
C23—H23A	0.9600	O2W—H2WB	0.7995
C23—H23B	0.9600	O2W—H2WA	0.9364
			
C2—C1—H1A	109.5	H23A—C23—H23C	109.5
C2—C1—H1B	109.5	H23B—C23—H23C	109.5
H1A—C1—H1B	109.5	C22—C24—C25	122.1 (3)
C2—C1—H1C	109.5	C22—C24—N3	118.7 (3)
H1A—C1—H1C	109.5	C25—C24—N3	119.2 (3)
H1B—C1—H1C	109.5	C27—C25—C24	117.7 (4)
C9—C2—C3	118.0 (3)	C27—C25—C26	120.4 (3)
C9—C2—C1	120.9 (4)	C24—C25—C26	121.9 (3)
C3—C2—C1	121.1 (4)	C25—C26—H26A	109.5
C4—C3—C2	121.9 (4)	C25—C26—H26B	109.5
C4—C3—H3D	119.1	H26A—C26—H26B	109.5
C2—C3—H3D	119.1	C25—C26—H26C	109.5
C3—C4—C6	118.0 (3)	H26A—C26—H26C	109.5
C3—C4—C5	119.8 (3)	H26B—C26—H26C	109.5
C6—C4—C5	122.1 (3)	C25—C27—C20	122.3 (4)
C4—C5—H5A	109.5	C25—C27—H27A	118.9
C4—C5—H5B	109.5	C20—C27—H27A	118.9
H5A—C5—H5B	109.5	C30—C28—C31	117.8 (3)
C4—C5—H5C	109.5	C30—C28—C29	120.6 (3)
H5A—C5—H5C	109.5	C31—C28—C29	121.6 (4)
H5B—C5—H5C	109.5	C28—C29—H29A	109.5
C7—C6—C4	122.0 (3)	C28—C29—H29B	109.5
C7—C6—N1	119.9 (3)	H29A—C29—H29B	109.5
C4—C6—N1	117.9 (3)	C28—C29—H29C	109.5
C6—C7—C9	117.3 (3)	H29A—C29—H29C	109.5
C6—C7—C8	122.8 (3)	H29B—C29—H29C	109.5
C9—C7—C8	119.9 (3)	C28—C30—C35	122.5 (3)
C7—C8—H8A	109.5	C28—C30—H30A	118.7
C7—C8—H8B	109.5	C35—C30—H30A	118.8
H8A—C8—H8B	109.5	C32—C31—C28	122.7 (3)
C7—C8—H8C	109.5	C32—C31—H31A	118.6
H8A—C8—H8C	109.5	C28—C31—H31A	118.6
H8B—C8—H8C	109.5	C31—C32—C34	117.8 (3)
C2—C9—C7	122.8 (3)	C31—C32—C33	120.7 (3)
C2—C9—H9A	118.6	C34—C32—C33	121.4 (3)
C7—C9—H9A	118.6	C32—C33—H33A	109.5
C11—C10—C17	122.7 (3)	C32—C33—H33B	109.5
C11—C10—N2	118.1 (3)	H33A—C33—H33B	109.5
C17—C10—N2	119.2 (3)	C32—C33—H33C	109.5
C13—C11—C10	117.2 (3)	H33A—C33—H33C	109.5
C13—C11—C12	119.9 (3)	H33B—C33—H33C	109.5
C10—C11—C12	122.9 (3)	C32—C34—C35	121.7 (3)
C11—C12—H12A	109.5	C32—C34—N4	118.6 (3)
C11—C12—H12B	109.5	C35—C34—N4	119.7 (3)
H12A—C12—H12B	109.5	C30—C35—C34	117.4 (3)
C11—C12—H12C	109.5	C30—C35—C36	119.8 (3)
H12A—C12—H12C	109.5	C34—C35—C36	122.8 (3)
H12B—C12—H12C	109.5	C35—C36—H36A	109.5
C14—C13—C11	122.6 (4)	C35—C36—H36B	109.5
C14—C13—H13A	118.7	H36A—C36—H36B	109.5
C11—C13—H13A	118.7	C35—C36—H36C	109.5
C13—C14—C16	117.9 (3)	H36A—C36—H36C	109.5
C13—C14—C15	120.2 (4)	H36B—C36—H36C	109.5
C16—C14—C15	121.8 (4)	C6—N1—H1D	109.5
C14—C15—H15A	109.5	C6—N1—H1E	109.5
C14—C15—H15B	109.5	H1D—N1—H1E	109.5
H15A—C15—H15B	109.5	C6—N1—H1F	109.5
C14—C15—H15C	109.5	H1D—N1—H1F	109.5
H15A—C15—H15C	109.5	H1E—N1—H1F	109.5
H15B—C15—H15C	109.5	C10—N2—H2A	109.5
C14—C16—C17	123.1 (3)	C10—N2—H2B	109.5
C14—C16—H16A	118.5	H2A—N2—H2B	109.5
C17—C16—H16A	118.5	C10—N2—H2C	109.5
C16—C17—C10	116.5 (3)	H2A—N2—H2C	109.5
C16—C17—C18	120.5 (3)	H2B—N2—H2C	109.5
C10—C17—C18	123.0 (3)	C24—N3—H3A	109.5
C17—C18—H18A	109.5	C24—N3—H3B	109.5
C17—C18—H18B	109.5	H3A—N3—H3B	109.5
H18A—C18—H18B	109.5	C24—N3—H3C	109.5
C17—C18—H18C	109.5	H3A—N3—H3C	109.5
H18A—C18—H18C	109.5	H3B—N3—H3C	109.5
H18B—C18—H18C	109.5	C34—N4—H4A	109.5
C20—C19—H19A	109.5	C34—N4—H4B	109.5
C20—C19—H19B	109.5	H4A—N4—H4B	109.5
H19A—C19—H19B	109.5	C34—N4—H4C	109.5
C20—C19—H19C	109.5	H4A—N4—H4C	109.5
H19A—C19—H19C	109.5	H4B—N4—H4C	109.5
H19B—C19—H19C	109.5	H1WA—O1W—H1WB	108.3
C21—C20—C27	117.7 (3)	H2WB—O2W—H2WA	113.3
C21—C20—C19	121.2 (4)	O8—S1—O5	111.70 (18)
C27—C20—C19	121.1 (4)	O8—S1—O6	108.90 (16)
C20—C21—C22	123.3 (3)	O5—S1—O6	108.93 (18)
C20—C21—H21A	118.4	O8—S1—O7	110.91 (17)
C22—C21—H21A	118.4	O5—S1—O7	108.42 (16)
C21—C22—C24	116.8 (3)	O6—S1—O7	107.89 (18)
C21—C22—C23	120.0 (3)	O3—S2—O1	111.13 (19)
C24—C22—C23	123.2 (3)	O3—S2—O4	111.42 (17)
C22—C23—H23A	109.5	O1—S2—O4	109.19 (15)
C22—C23—H23B	109.5	O3—S2—O2	109.28 (16)
H23A—C23—H23B	109.5	O1—S2—O2	108.40 (17)
C22—C23—H23C	109.5	O4—S2—O2	107.31 (18)

### Hydrogen-bond geometry (Å, °)

**Table:**

*D*—H···*A*	*D*—H	H···*A*	*D*···*A*	*D*—H···*A*
N1—H1D···O6	0.89	1.81	2.668 (4)	162.
N1—H1D···S1	0.89	2.74	3.603 (3)	164.
N2—H2B···O1W	0.89	2.12	2.834 (4)	137.
N2—H2C···O4	0.89	1.88	2.763 (4)	173.
N2—H2C···S2	0.89	3.00	3.861 (3)	162.
N3—H3A···O7	0.89	1.89	2.761 (4)	166.
N3—H3B···O2W	0.89	2.12	2.877 (4)	142.
N4—H4A···O7	0.89	1.97	2.800 (4)	155.
N3—H3A···S1	0.89	3.05	3.858 (3)	153.
O1W—H1WA···O1	0.78	2.02	2.782 (4)	165.
O1W—H1WB···O5	0.97	1.82	2.788 (4)	173.
O1W—H1WB···S1	0.97	2.88	3.805 (3)	159.
O2W—H2WB···O5	0.80	1.97	2.756 (4)	166.
O2W—H2WB···S1	0.80	2.99	3.761 (3)	162.
N1—H1E···O2W^i^	0.89	1.99	2.837 (4)	158.
N1—H1F···O4^ii^	0.89	1.99	2.807 (4)	153.
N1—H1F···S2^ii^	0.89	2.92	3.622 (3)	137.
N2—H2A···O6^iii^	0.89	1.85	2.735 (4)	171.
N2—H2A···S1^iii^	0.89	3.01	3.800 (3)	149.
N3—H3C···O2^iv^	0.89	1.81	2.695 (4)	178.
N3—H3C···S2^iv^	0.89	2.96	3.788 (3)	156.
N4—H4B···O1W^iv^	0.89	1.97	2.842 (4)	165.
N4—H4C···O2^ii^	0.89	1.79	2.683 (4)	178.
N4—H4C···S2^ii^	0.89	2.81	3.616 (3)	151.
O2W—H2WA···O1^v^	0.94	1.86	2.781 (4)	168.
O2W—H2WA···S2^v^	0.94	2.88	3.791 (3)	165.

Symmetry codes: (i) *x*−1, *y*, *z*; (ii) −*x*, −*y*+1, *z*+1/2; (iii) −*x*+1, −*y*+1, *z*−1/2; (iv) −*x*+1, −*y*+1, *z*+1/2; (v) *x*+1, *y*, *z*.
